# Rationally Designed Antibodies as Research Tools to Study the Structure–Toxicity Relationship of Amyloid-β Oligomers

**DOI:** 10.3390/ijms21124542

**Published:** 2020-06-25

**Authors:** Ryan Limbocker, Benedetta Mannini, Rodrigo Cataldi, Shianne Chhangur, Aidan K. Wright, Ryan P. Kreiser, J. Alex Albright, Sean Chia, Johnny Habchi, Pietro Sormanni, Janet R. Kumita, Francesco S. Ruggeri, Christopher M. Dobson, Fabrizio Chiti, Francesco A. Aprile, Michele Vendruscolo

**Affiliations:** 1 Centre for Misfolding Diseases, Department of Chemistry, University of Cambridge, Cambridge CB2 1EW, UK; bm475@cam.ac.uk (B.M.); rlc67@cam.ac.uk (R.C.); sc2066@cam.ac.uk (S.C.); skrc2@cam.ac.uk (S.C.); jh884@cam.ac.uk (J.H.); ps589@cam.ac.uk (P.S.); jrk38@cam.ac.uk (J.R.K.); fsr26@cam.ac.uk (F.S.R.); md44@cam.ac.uk (C.M.D.); 2 Department of Chemistry and Life Science, United States Military Academy, West Point, NY 10996, USA; aidan.wright@westpoint.edu (A.K.W.); ryan.kreiser@westpoint.edu (R.P.K.); james.albright@westpoint.edu (J.A.A.); 3 Department of Experimental and Clinical Biomedical Science, University of Florence, 50134 Florence, Italy; fabrizio.chiti@unifi.it

**Keywords:** protein misfolded oligomers, structure–toxicity relationship, rationally designed antibodies, research tools, biophysics, amyloid-β, Alzheimer’s disease

## Abstract

Alzheimer’s disease is associated with the aggregation of the amyloid-β peptide (Aβ), resulting in the deposition of amyloid plaques in brain tissue. Recent scrutiny of the mechanisms by which Aβ aggregates induce neuronal dysfunction has highlighted the importance of the Aβ oligomers of this protein fragment. Because of the transient and heterogeneous nature of these oligomers, however, it has been challenging to investigate the detailed mechanisms by which these species exert cytotoxicity. To address this problem, we demonstrate here the use of rationally designed single-domain antibodies (DesAbs) to characterize the structure–toxicity relationship of Aβ oligomers. For this purpose, we use Zn^2+^-stabilized oligomers of the 40-residue form of Aβ (Aβ_40_) as models of brain Aβ oligomers and two single-domain antibodies (DesAb_18-24_ and DesAb_34-40_), designed to bind to epitopes at residues 18–24 and 34–40 of Aβ_40_, respectively. We found that the DesAbs induce a change in structure of the Zn^2+^-stabilized Aβ_40_ oligomers, generating a simultaneous increase in their size and solvent-exposed hydrophobicity. We then observed that these increments in both the size and hydrophobicity of the oligomers neutralize each other in terms of their effects on cytotoxicity, as predicted by a recently proposed general structure–toxicity relationship, and observed experimentally. These results illustrate the use of the DesAbs as research tools to investigate the biophysical and cytotoxicity properties of Aβ oligomers.

## 1. Introduction

The development and progression of Alzheimer’s disease (AD) is characterized by the misfolding and aggregation of the amyloid-β peptide (Aβ), which eventually results in the formation of dense senile plaques rich in fibrillar Aβ aggregates [1,2,3]. Increasing evidence suggests that the oligomeric aggregates which form as intermediates during the Aβ deposition process, rather than mature fibrils, are the predominant species capable of inducing neuronal dysfunction [4,5,6,7]. Targeting the rates of formation or the physicochemical properties of Aβ oligomers, therefore, represents one of the most promising therapeutic approaches to the treatment of AD [8,9].

Intense focus on inhibiting Aβ deposition has resulted in the characterization of numerous molecules, including small molecules, antibodies and molecular chaperones, which are able to prevent Aβ aggregation, and therefore, reduce the rate of Aβ oligomer formation, although no disease-modifying compound has yet been approved for clinical use [10,11]. These failures are, in part, attributable to the inclusion in clinical trials of participants without Aβ-related pathologies, thus confounding the analysis, and of participants with advanced AD, when the amyloid burden has already reached an advanced stage, making potential drugs against Aβ-induced neuronal damage less effective [12,13,14]. They are also a consequence of poor quantitative endpoints regarding the impact of candidate inhibitors on the microscopic steps governing Aβ aggregation, including the number of the highly cytotoxic oligomeric species produced in the presence of potential therapeutics [15,16,17,18,19,20,21], as well as an incomplete mechanistic understanding of the impact of candidate compounds on the physicochemical properties of oligomeric species and their closely related ability to induce cytotoxicity.

Given the advantages of using antibodies as research tools [22,23], in particular for their very high selectivity for a specific target (e.g., epitopes on the surface of Aβ oligomers) [18,24,25], in addition to reports that have illustrated the efficacy of anti-Aβ immunotherapies [9,26] and the potential of anti-Aβ antibodies for early diagnosis [27,28], we recently focused on the development of rationally designed antibodies to inhibit the aggregation of Aβ through the selective targeting of specific microscopic steps inherent to its self-assembly and propagation [18]. Of particular note, we previously found that the designed single-domain antibody raised against residues 18–24 of Aβ (DesAb_18-24_) is a potent inhibitor of the microscopic step of primary nucleation of the Aβ_42_ amyloid fibril formation process. Moreover, its administration to a *C. elegans* model of AD was shown to reduce the toxicity implicit to Aβ_42_ aggregation when administered before or during the development of a pathological phenotype [18]. In light of these observations, and to contribute new tools with which to study the impact of candidate therapeutic compounds on the physicochemical properties of intermediate oligomeric species, we sought to assess the impact of this and a second rationally designed single-domain antibody raised against residues 34–40 of Aβ_40_ (DesAb_34-40_) on stabilized cytotoxic oligomeric species of Aβ_40_ after their formation, which were generated from both primary and secondary nucleation pathways in the endogenous Aβ aggregation reaction [19].

To investigate the relationship between the structure and the toxicity of Aβ_40_ oligomers, we first determined the effects of DesAb_18-24_ and DesAb_34-40_ on the in vitro aggregation of Aβ_40_. Then, we analyzed the effects of these DesAbs on the size and hydrophobicity of Zn^2+^-stabilized Aβ_40_ oligomers formed under near physiological conditions. We found that the DesAbs induced the formation of larger and more hydrophobic Aβ_40_ oligomers. As a control, we then showed that the natural product trodusquemine, which protects cell membranes from the cytotoxic effects of protein misfolded oligomers at substoichiometric concentrations [29], induces similar effects to these DesAbs when used at concentrations that are superstoichiometric and higher than those normally used in cells or patients due to its intrinsic toxicity. Increases in size and hydrophobicity are known to cause a decrease and increase of oligomer toxicity, respectively [30,31,32,33,34,35,36]. In fact, consistent with previous quantifications of the size–hydrophobicity relationship modulated by mutations on another protein system [30], we observed that these effects mediated by our DesAbs on Aβ_40_ did not significantly change the degree of cytotoxicity of the oligomers towards SH-SY5Y neuroblastoma cells, indicating that the simultaneous increase in oligomer size and hydrophobicity produces antagonistic effects on oligomer toxicity.

## 2. Results

### 2.1. The DesAbs Inhibit Aβ_40_ Aggregation

In this work, we investigate two computationally-designed, single-domain antibodies, called DesAb_18-24_ and DesAb_34-40_, which target the regions 18–24 and 34–40 of Aβ_40_, respectively. These antibodies were generated by grafting a complementary peptide into the third complementary determining region (CDR3) of a single-domain antibody scaffold, as described previously [18,37]. 

**Figure 1:**
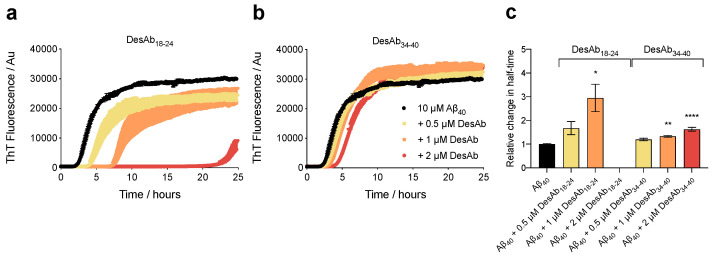
DesAb_18-24_ and DesAb_34-40_ inhibit Aβ_40_ aggregation. (**a**,**b**) Thioflavin T (ThT) fluorescence intensity profiles for the aggregation of Aβ_40_ (10 μM) incubated in the absence (black) and presence of 0.5 μM (yellow), 1 µM (orange) and 2 μM (red) DesAb_18-24_ (**a**) and DesAb_34-40_ (**b**). Upward error bars indicate standard error of the mean (SEM) of three technical replicates. (**c**) Relative change in half-time (t_1/2_) calculated from dividing t_1/2_ for each experimental condition by the average t_1/2_ determined for Aβ_40_ in the absence of DesAbs to show the relative delay in aggregation induced by the antibodies. Error bars indicate SEM of three technical replicates. Individual antibody groups were analyzed by one-way ANOVA followed by a Bonferroni’s multiple comparison test relative to Aβ_40_ in buffer. The symbols *, **, and **** indicate *p* < 0.05, 0.01 and 0.0001, respectively.

We first investigated the effect of these two DesAbs on Aβ_40_ aggregation. Samples containing monomeric Aβ_40_ at a concentration of 10 µM (20 mM Tris, 100 mM NaCl, pH 7.4) were prepared in the absence and presence of 20-, 10-, and 5-fold substoichiometric concentrations of the designed antibodies and incubated under quiescent conditions at 37 °C. The fibrilization process was monitored by means of thioflavin T fluorescence (ThT) in the presence of 20 µM ThT. We observed that DesAb_18-24_ strongly inhibits Aβ_40_ aggregation, causing a significant delay in the half-time of aggregation (t_1/2_) (Figure 1a), which is in agreement with previous findings for Aβ_42_ [18]. Indeed, at the highest concentration of DesAb_18-24_ (2 µM), Aβ_40_ did not aggregate beyond t_1/2_ in this experiment on the time course of 25 h. Further experiments on DesAb_34-40_ revealed that this designed antibody was also able to interfere with the reaction, as observed by an overall delay in the aggregation kinetics (Figure 1b). Interestingly, both DesAbs inhibited the aggregation kinetics in a concentration-dependent manner, as illustrated by the relative changes in t_1/2_ (Figure 1c). Experiments were carried out in triplicate, and indicated that both designed antibodies can significantly delay Aβ_40_ aggregation. 

### 2.2. The DesAbs Bind Stabilized Aβ_40_ Oligomers and Increase Their Hydrophobic Surface Area

During the aggregation of Aβ_40_ at the concentrations used here, the peak oligomer concentration reached circa 1% of the total monomer concentration at the half-point of the aggregation reaction [21,38]. Given the very low abundance and high instability of on-pathway oligomers, numerous methods have been described to generate and stabilize oligomers at concentrations that are orders of magnitude higher [39,40,41,42]. For this purpose, oligomers of Aβ_40_ stabilized by Zn^2+^ were produced as previously described [42]. These oligomers are stabilized by a metal ion that is naturally present at the synapse, and may therefore resemble those that exist in AD [42].

**Figure 2:**
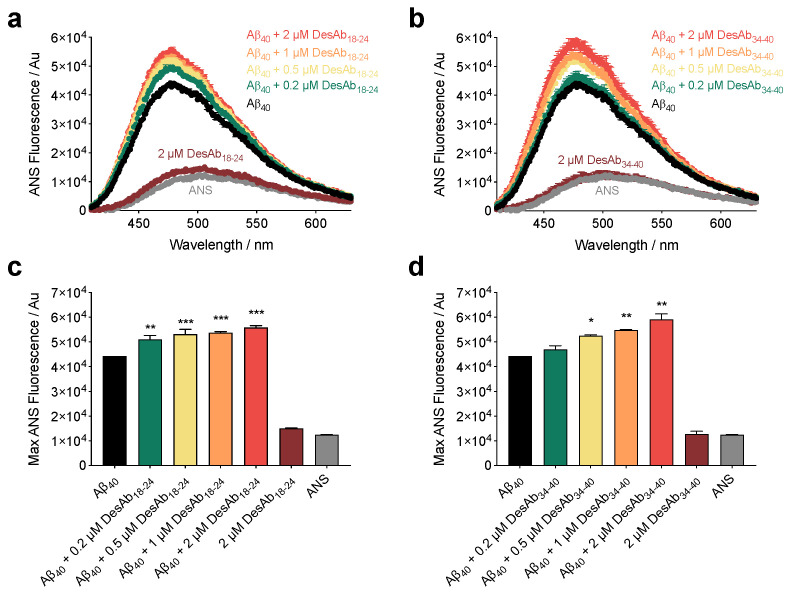
DesAb_18-24_ and DesAb_34-40_ increase the hydrophobic area of stabilized Aβ_40_ oligomers. ANS binding measurements for oligomers of Aβ_40_ incubated at a concentration of 10 µM in the absence (black) or presence of 0.2 μM (green), 0.5 μM (yellow), 1 µM (orange) and 2 μM (red) DesAb_18-24_ (**a**) and DesAb_34-40_ (**b**). The intensity of maximum ANS fluorescence for DesAb_18-24_ (**c**) and DesAb_34-40_ (**d**), determined from the spectra indicated in (**a**) and (**b**), respectively. Note that the highest concentration of DesAbs tested (2 µM, dark red) did not significantly change the fluorescence of free ANS (grey) in the absence of oligomers. Error bars denote SEM of duplicate spectra. Samples containing oligomers were analyzed by one-way ANOVA followed by a Bonferroni’s multiple comparison test relative to oligomers in buffer. The symbols *, **, and *** indicate *p* < 0.05, 0.01 and 0.001, respectively.

We first tested the ability of these antibodies to bind to stabilized Aβ_40_ oligomers using a dot-blot assay (Supplementary Figure S1). Then, we evaluated the degree of hydrophobicity of the oligomers in the absence and presence of the DesAbs using the fluorescent dye 8-anilinonapthalene-1-sulfonic acid (ANS), which binds to hydrophobic patches on the surfaces of protein aggregates, generating a quantum yield increase and blue shift of fluorescence relative to unbound dye [43]. We incubated stabilized Aβ_40_ oligomers after their formation [42] for 2 h at 20 °C at a concentration of 10 µM (in monomer equivalents) in 20 mM Tris, 100 mM NaCl and at pH 7.4 in the absence and presence of increasing concentrations (0.2 µM, 0.5 µM, 1 µM, and 2 µM) of each antibody. After incubation, ANS was added to a final concentration of 30 µM (i.e., 3-fold excess). Similar to our previous characterization for Aβ_42_ oligomers [29], ANS fluorescence intensity was observed to increase and undergo a blue-shift from ca. 510 nm to ca. 475 nm in the presence of stabilized Aβ_40_ oligomers. Incubation in the presence of increasing concentrations of DesAb_18-24_ and DesAb_34-40_ resulted in the amplification of this effect with a clear, well-defined dose dependence (Figure 2a,b). Data were quantified by maximum ANS fluorescence intensity (Figure 2c,d). Representative data are shown for three independent protein preparations that yielded consistent results. The incubation of 2 µM DesAbs (i.e., the highest ratio tested) in the absence of oligomers exerted a negligible effect on ANS fluorescence. These results indicate that the hydrophobicity of the Aβ_40_ oligomers increased as a function of antibody concentration.

### 2.3. DesAbs Increase the Size of Stabilized Aβ_40_ Oligomers

**Figure 3:**
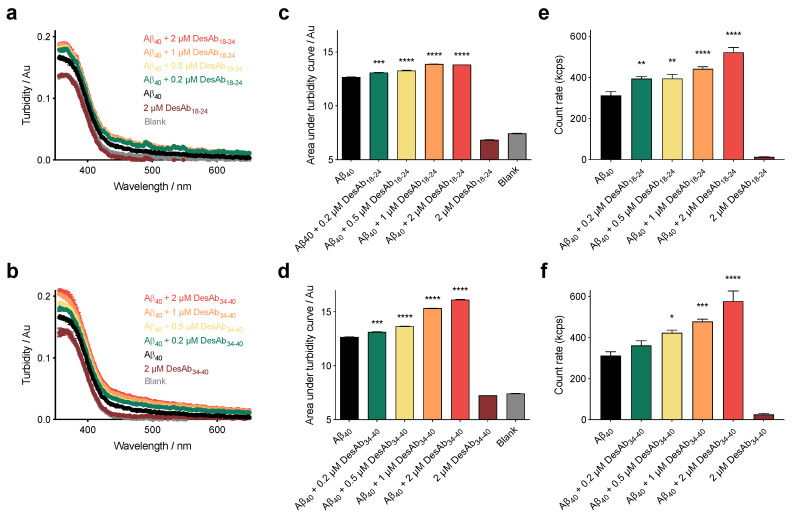
DesAb_18-24_ and DesAb_34-40_ increase the size of stabilized Aβ_40_ oligomers. (**a**,**b**) Turbidity measurements for oligomers of Aβ_40_ incubated at a concentration of 10 µM in the absence (black) or presence of 0.2 μM (green), 0.5 μM (yellow), 1 µM (orange) and 2 μM (red) of DesAb_18-24_ (**a**) and DesAb_34-40_ (**b**). Samples are identical to those shown in Figure 2. (**c**,**d**). Turbidity traces analyzed by area under the curve integration. Note that the highest concentration of DesAbs tested (2 µM, dark red) did not significantly change the absorbance relative to the blank (ANS, grey). Error bars denote SEM of duplicate spectra. (**e**,**f**) SLS measurements in the absence of ANS and the presence of Aβ_40_ oligomers and increasing concentrations of the designed antibodies. Note negligible scattering from the highest ratio of the DesAbs tested in the absence of oligomers. Error bars denote SEM of six measurements. In all panels, the symbols *, **, *** and **** indicate *p* < 0.05, 0.01, 0.001 and 0.0001, respectively. Samples containing oligomers were analyzed by one-way ANOVA followed by Bonferroni’s multiple comparison test relative to oligomers in buffer.

We hypothesized that DesAb_18-24_ and DesAb_34-40_ may be able to sequester the aggregates in solution via a clustering-based mechanism. This mechanism has been described for molecular chaperones in the presence of several oligomeric species comprising various peptides and proteins [31,32,33,35]. To probe this interaction, we first measured the turbidimetry absorbance (turbidity) of the same samples that were investigated in the ANS experiment. In parallel with an increase in hydrophobicity, we also observed that the spectra were shifted to higher values of turbidity as a function of antibody concentration (Figure 3a,b). Data were quantified by integrating the area under each curve (Figure 3c,d). The results shown are representative of three independent experiments that gave consistent results. It should be noted that the highest concentration of DesAbs used in our experiments absorbed at a level which cannot recapitulate the full effects observed on size in the presence of the oligomers, indicating that the antibody exerts a negligible effect on absorbance in the absence of oligomers. These results demonstrate that the size of the oligomers increased in a dose-dependent manner by DesAb_18-24_ and DesAb_34-40_.

In order to validate the increase in size with an ANS-free independent method, samples were incubated under the same conditions and measured using static light scattering (SLS). In agreement with the turbidity measurements, we found that the incubation of the stabilized oligomers in the presence of increasing concentrations of DesAbs resulted in augmented light scattering (Figure 3e,f). As additional evidence of a size increase of the aggregates in solution, we also employed sedimentation-based dot-blot assays. In these measurements, larger aggregates are postulated to sediment more readily than unmodified small oligomers. Oligomers were incubated as previously described with and without antibodies, centrifuged (13,000 rcf, 20 °C, 15 min), concentrated during resuspension, and deposited atop a nitrocellulose membrane. Minimal protein was deposited in the samples containing oligomers in the absence of antibodies, as recognized using the anti-Aβ antibody 6E10. The presence of the DesAbs, however, resulted in an increase of Aβ_40_ sedimentation with dose dependence (Supplementary Figure S2). Taken together, the turbidimetry, SLS, and sedimentation measurements support the conclusion that the DesAbs significantly increase the size of the Aβ_40_ oligomers in solution. We note that recent work on oligomers demonstrated that protein misfolded oligomer toxicity is independent of their secondary structures [44], and we therefore elected to focus on the size–hydrophobicity–toxicity relationship in detail.

**Figure 4:**
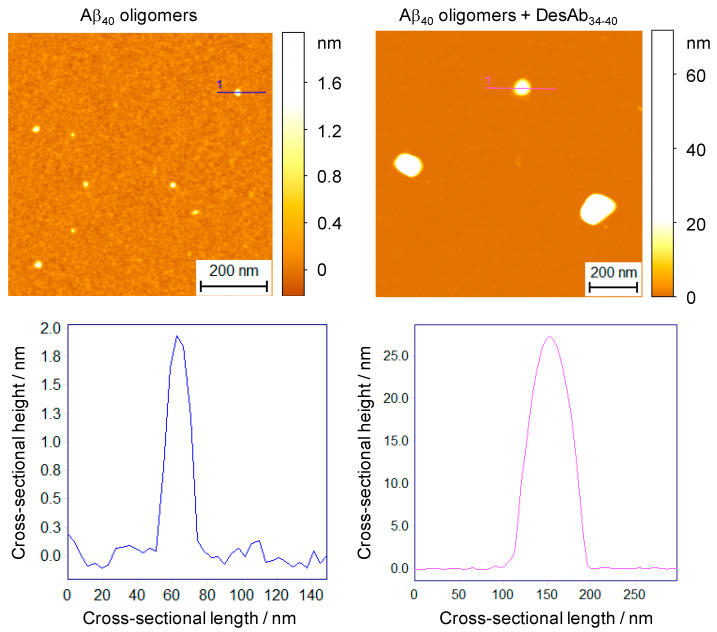
Phase-controlled AFM measurements of the oligomer-antibody complexes. Aβ_40_ oligomers were incubated at a concentration of 10 µM in the absence (left) and presence of 2 µM DesAb_34-40_ (right) and diluted 20-fold in buffer before deposition. Large oligomeric clusters (white aggregates in the 3D AFM maps) were observed in the presence of DesAb_34-40_. Representative aggregate heights are shown below each 3D color plot for the indicated cross-sectional profiles. Scale bars, 0.2 µm.

To directly visualize the size and morphology of these oligomer clusters, we next investigated the oligomer-DesAb_34-40_ complex by phase-controlled atomic force microscopy (AFM) [45,46]. Stabilized Aβ_40_ oligomers were incubated as previously described at a concentration of 10 µM (in monomer equivalents) in the absence and presence of 2 µM DesAb_34-40_ (2 h, 20 °C), and diluted in buffer by a factor of 20 before conventional, manual deposition atop an atomically flat mica surface. In the absence of DesAb_34-40_, oligomers were 1–5 nm in height (Figure 4), in agreement with previous characterizations [42]. In the presence of DesAb_34-40_, clusters ranging in cross-sectional height from 20 to 50 nm were readily observed, in good agreement with the size results observed in the bulk experiments (Figure 4).

### 2.4. Trodusquemine Binds Stabilized Aβ_40_ Oligomers and Increases their Hydrophobic Surface Area

The natural product trodusquemine has been characterized for its ability to displace protein misfolded oligomers from the surfaces of cell membranes, resulting in a marked decrease in oligomer-induced cytotoxicity towards neuroblastoma cells. Its protective effects were observed at physiological concentrations of this aminosterol, corresponding to equimolar or substoichiometric concentrations of the molecule relative to oligomers, where greater than equimolar concentrations of trodusquemine demonstrated its intrinsic toxicity to cells [29]. Further, we previously observed that trodusquemine can increase the size and hydrophobicity of Aβ_42_ oligomers at superstoichiometric concentrations, whereas the effects were not significant at substoichiometric concentrations [29]. 

**Figure 5:**
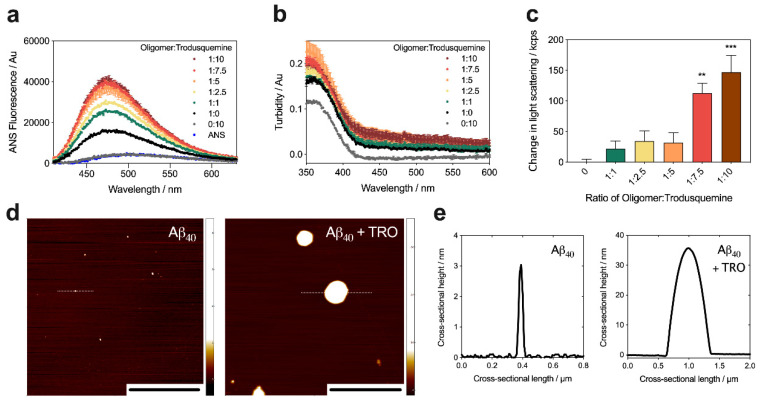
Trodusquemine increases the hydrophobicity and size of stabilized Aβ_40_ oligomers. (**a**,**b**) Oligomers were incubated at a concentration of 5 µM in the absence (black) and presence of 5 µM (green), 12.5 µM (yellow), 25 µM (orange), 37.5 µM (red), and 50 µM (brown) trodusquemine and monitored for the binding of ANS (**a**) and turbidity absorbance (**b**). Grey traces indicate the effect of 50 µM trodusquemine (TRO) (i.e., the highest ratio tested) in the absence of oligomers. Error bars denote SEM of duplicate spectra. (**c**) SLS measurements after oligomer incubation in the presence of increasing concentrations of trodusquemine. Data are shown as the change in light scattered. Error bars represent the SEM of three replicates, and the symbols ** and *** indicate *p* < 0.01 and 0.001, respectively. Samples containing oligomers were analyzed by one-way ANOVA followed by Bonferroni’s multiple comparison test relative to oligomers in buffer. (**d**) AFM maps for oligomers incubated in the absence (left panel) or presence of a 5-fold excesses of trodusquemine (right panel). Scale bars, 2 µm. Upper limits on the color scales are circa 4.2 nm for Aβ_40_ oligomers alone (left panel) and 35 nm for the Aβ_40_ oligomer-trodusquemine complex (right panel). (**e**) Representative cross-sectional heights of oligomers in the absence (left panel) and presence of trodusquemine (right panel). Dotted white lines in indicate the corresponding sampled cross section from (**d**).

To build on this result and to increase the size and hydrophobicity of Aβ_40_ oligomers similarly to our DesAbs, and thus to obtain a control case for this study, we incubated stabilized Aβ_40_ oligomers at a concentration of 5 µM (in monomer equivalents) in 20 mM Tris, 100 mM NaCl, pH 7.4, in the absence and presence of increasing concentrations of trodusquemine (0–50 µM, 1 h at 20 °C). After incubation, ANS was added to a final concentration of 15 µM (i.e., 3-fold excess). The incubation of Aβ_40_ oligomers with trodusquemine increased the maximum fluorescence intensity and caused a further blue-shift of its wavelength, with a well-defined dose dependence (Figure 5a), indicating a prominent increase in solvent-exposed hydrophobicity. As a control, both the intensity and maximum wavelength of free ANS were unchanged in the absence of oligomers and in the presence of the highest concentration of trodusquemine (i.e., 50 µM). 

The incubation of stabilized Aβ_40_ oligomers with trodusquemine and ANS also resulted in a well-defined increase in turbidity with a clear dose dependence (Figure 5b). Using SLS measurements in the absence of ANS, we also observed a dose-dependent increase in the extent of light scattered upon the incubation of the oligomers in the presence of increasing concentrations of trodusquemine (Figure 5c). Negligible turbidity and light scattering were measured for 50 µM trodusquemine in the absence of oligomers (Figure 5b,c), indicating that the molecule does not form large clusters under these conditions, and does not contribute significantly to the observations in the presence of Aβ_40_ oligomers. The relative change in the extent of light scattered upon the interaction of increasing concentrations of trodusquemine with the oligomers is in good agreement with the turbidity data. 

The trodusquemine-mediated modification of stabilized Aβ_40_ oligomers was also studied using phase-controlled AFM [45,47,48]. Sample deposition was carried out using a recently developed microfluidic spraying device [49]. The utilization of this methodology affords the advantage of spraying microdroplets atop the mica substrate at subpicoliter volumes, which evaporate on the timescale of milliseconds, thereby avoiding artefacts due to surface mass transport processes caused by drying [49], which are particularly likely while drying significant concentrations of a polyvalent small molecule in addition to oligomers. Stabilized Aβ_40_ oligomers were incubated as previously described at a concentration of 5 µM in the absence and presence of a 5-fold excess of trodusquemine (1 h, 20 °C) and subsequently diluted by a factor of five in buffer prior to spraying atop an atomically flat mica surface (Figure 5d). In the absence of molecules, the cross-sectional heights of the oligomers were typically 1–5 nm in height, in agreement with previous reports [42]. Upon the addition of a 5-fold excess of molecule, the presence of large clustered species was observed with cross-sectional heights ranging from approximately 20 to 40 nm (Figure 5e), in good agreement with the turbidity and SLS results observed in the bulk experiments.

### 2.5. The DesAbs do not Change the Toxicity of Aβ_40_ Oligomers for Human Neuroblastoma Cells

**Figure 6:**
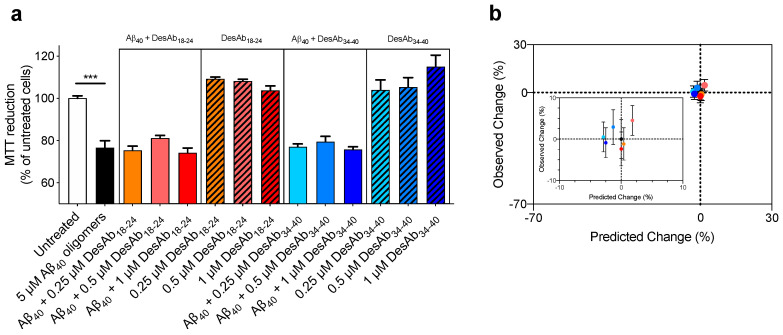
The interaction of the DesAbs with stabilized Aβ_40_ oligomers does not change their toxicity. (a) Stabilized oligomers of Aβ_40_ were resuspended in the cell culture medium at a concentration of 5 μM and incubated in the absence (black plain bar) or presence of increasing concentrations (0.25, 0.5 and 1 µM) of DesAb_18-24_ (red plain bars) and DesAb_34-40_ (blue plain bars) for 1 h at 37 °C and then added to the cell culture medium of SH-SY5Y cells for 24 h. The cells were also treated with corresponding concentrations of the antibodies pre-incubated in the absence of oligomers (dashed bars with corresponding colors). Error bars indicate SEM of six replicates. The symbol *** indicates *p* < 0.001 (unpaired, two-tailed Student’s *t*-test). All conditions containing both antibodies and oligomers were not significantly different in comparison to cells treated with oligomers alone (individual antibody groups were compared by one-way ANOVA using Bonferroni’s multiple comparisons relative to cells treated with oligomers alone). (b) The wavelength of maximum ANS fluorescence as a probe of solvent exposed hydrophobicity and the Rayleigh ratios from SLS measurements as a measure of size were used to predict the change in MTT reduction caused by oligomers in the absence and presence of the various concentrations of DesAbs (Supplementary Table S1) as previously described [30]. The predicted values were plotted against the observed ones from (a). The formula predicts that the DesAb-mediated increase in both hydrophobicity and size of Aβ_40_ oligomers does not significantly change the toxicity, as was observed experimentally.

To assess whether the increase in oligomer size and hydrophobicity induced by DesAbs and trodusquemine resulted in changes of oligomer toxicity, cell viability experiments using the MTT reduction assay were performed using SH-SY5Y human neuroblastoma cells in the presence of the oligomers. Stabilized Aβ_40_ oligomers (5 µM, in monomer equivalents) were formed and incubated in the absence and presence of increasing concentrations of DesAb_18-24_ and DesAb_34-40_ (0.25 µM, 0.5 µM and 1 µM) for 1 h at 37 °C, and subsequently added to the cell culture medium of SH-SY5Y cells for 24 h at 37 °C. These concentrations were selected to match the biophysical experiments. The cells were also treated with the corresponding concentrations of antibodies in the absence of oligomers, which was not observed to induce deleterious changes in cellular viability. Aβ_40_ oligomers were found to decrease the viability of the cells by 23.4 ± 3.6%, and this toxicity was not changed significantly upon the addition of either DesAb when co-incubated with oligomers at these concentrations (Figure 6a).

**Figure 7:**
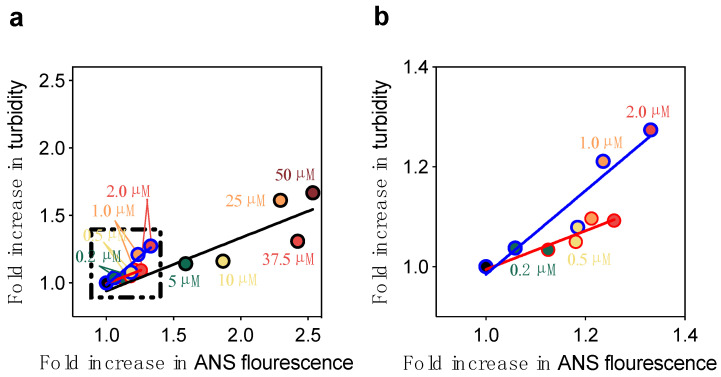
Size-hydrophobicity relationship for increasing concentrations of the designed antibodies and trodusquemine. (**a**) Linear fits between size and solvent-exposed hydrophobicity for increasing concentrations of DesAb_34-40_ (blue line, slope = 0.84 ± 0.15, R = 0.95), DesAb_18-24_ (red, slope = 0.38 ± 0.08, R = 0.86), and trodusquemine (TRO, black line, slope = 0.39 ± 0.12, R = 0.86). (**b**) Zoomed in view of panel **a** for the designed antibodies.

We then compared the observed experimental changes in cell viability upon the addition of various concentrations of DesAbs to Aβ_40_ oligomers to the predicted changes, where the latter was estimated using a previously defined formula [30] (Figure 6b and Supplementary Table S1). The comparison indicated that the toxicity of stabilized Aβ_40_ oligomers was neither predicted nor observed to change upon the addition of these concentrations of DesAb_18-24_ and DesAb_34-40_. This might appear surprising, as oligomer size and hydrophobicity are known to be key determinants of oligomer toxicity [30,31,32,33,34,35,36]. However, increases in size and hydrophobicity are predicted to decrease and increase oligomer toxicity, respectively, resulting in counteracting effects and no overall change in toxicity. The highly significant correlations found between Aβ_40_ oligomer size and hydrophobicity in the presence of designed antibodies and trodusquemine indicate that these agents cause paired increases of both parameters (Figure 7), resulting in no significant effect on the toxicity of the Aβ_40_ oligomers (Supplementary Table S1).

We also measured the toxicity of the oligomers with a greater concentration (i.e., 10 µM, monomer equivalents) and at the same molar ratios studied above, i.e., 0.5 µM, 1 µM and 2 µM antibodies (Supplementary Figure S3). Again, the addition of DesAb_18-24_ did not alter the toxicity of the stabilized Aβ_40_ oligomers, suggesting that the concentration of toxic species is not likely to result in different biological activity of the antibodies. Collectively, these results suggest that the ability of the antibodies to cluster the oligomers into larger and more hydrophobic species does not change the toxicity of the aggregates, which agrees well with the predictions (Supplementary Table S1). 

It was not possible to carry out the MTT assays for Aβ_40_ oligomers incubated with trodusquemine at the superstoichiometric concentrations that are necessary to observe a significant clustering effect of the oligomers [29]. Indeed, the molecule alone was shown to be toxic to cells at concentrations greater than a few micromolar [29]. Such an experiment would further fail to account for the unique ability of trodusquemine to bind to cell membranes and displace Aβ oligomers.

## 3. Discussion

Aβ oligomers are highly cytotoxic species associated with the aggregation reaction of Aβ, which is central to the onset and development of AD [4,5,6,7]. Extensive past work has been carried out to investigate the structure-toxicity relationship of these oligomers, for example through the use of cross-linking to generate stabilized oligomeric forms [50,51,52]. More specifically, the toxicity of aberrant oligomeric protein aggregates has been previously described as a function of size and hydrophobicity for multiple proteins, where the most cytotoxic aggregates are generally small and have extensive hydrophobic surface exposure [30,32,35]. These physiochemical properties play a central role in the ability of oligomers to cause cellular dysfunction, which can result from the uptake of the oligomeric proteins into the cell [53,54,55], or directly by the oligomer-induced perturbation of cell membranes [3,36]. Here, we utilized rationally designed antibodies (DesAbs) to probe the structure–toxicity relationship of Zn^2+^-stabilized Aβ_40_ oligomers, which we adopted as models of the Aβ oligomers that may form in synaptic regions where Zn^2+^ tends to be abundant [42]. We used these DesAbs as molecular tools, following our previous illustrations of their applications for diagnostic [27] or therapeutic purposes [18]. 

An increase in size alone, in the absence of other physicochemical changes, has been well documented to suppress oligomer cytotoxicity. Indeed, high concentrations of molecular chaperones, including clusterin and aβ-crystallin, have been shown to reduce the cellular toxicity of oligomers comprised of Aβ_42_, HypF-N, and islet amyloid polypeptide through a clustering-based mechanism [31,33,35]. The reduction in toxicity mediated by clustering was therefore attributed to a prominent decrease in the surface to volume ratio of the aggregates, thereby reducing the diffusibility of the oligomers and limiting their interaction with the cellular membrane [32,35]. In the case of the DesAb_18-24_ and DesAb_34-40_, and trodusquemine at superstoichiometric concentrations, Aβ_40_ oligomer size was significantly increased upon interaction with the antibodies and the aminosterol.

In addition to size, hydrophobicity is a key determinant of oligomer cytotoxicity [34,36]. A decrease in hydrophobicity is associated with lower cytotoxicity. At superstoichiometric concentrations of clusterin and aβ-crystallin relative to HypF-N oligomers, these molecular chaperones were shown to be able to mask the solvent exposure of protein misfolded oligomers in the absence of significant oligomer size increases [32,35]. The corresponding decrease in cellular toxicity was explained by their lessened affinity for cell membranes induced by the lowering of oligomer solvent-exposed hydrophobicity. For DesAb_18-24_ and DesAb_34-40_ and trodusquemine at superstoichiometric concentrations, however, Aβ_40_ oligomer hydrophobicity was significantly increased upon interaction with the antibodies and molecules. 

**Figure 8:**
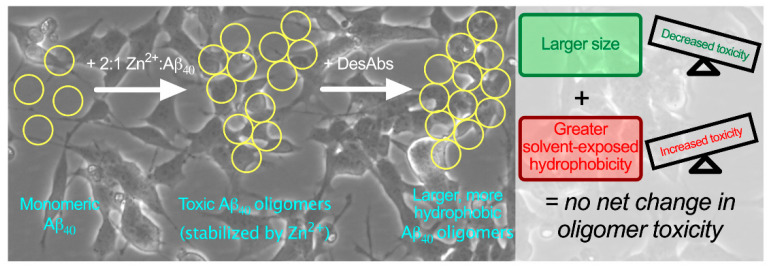
We have used rationally designed antibodies (DesAbs) as molecular tools to investigate the size-hydrophobicity-toxicity relationship of Aβ_40_ oligomers. Incubation with DesAbs increased the size and hydrophobicity of Zn^2+^-stabilized Aβ_40_ oligomers. These physicochemical changes decrease and increase the toxicity of these particular model oligomers, respectively, resulting in no net overall change in their toxicity upon administration to cultured human neuroblastoma cells.

The DesAbs modified the structure of the oligomers in a way that is expected to increase their cytotoxicity by increasing their hydrophobicity. At the same time, the DesAbs could also be expected to decrease the toxicity of the oligomers by increasing their size. Thus, by using the DesAbs as molecular tools, we leveraged a well-described size–hydrophobicity formula [30] to predict that these physiochemical changes would cancel each other in terms of the resulting toxicity of the oligomers. Experimentally, we confirmed these predictions by observing that these changes offset each other, as manifested by negligible changes in oligomer toxicity to the neuroblastoma cells upon interaction with the DesAbs (Figure 8). 

Trodusquemine and other molecules within the aminosterol family possess the unique ability to bind to cell membranes and prevent the deleterious association of oligomers via a displacement-based mechanism [29,56,57]. Given the absence of a protective effect of the DesAbs toward neuroblastoma cells when incubated alongside oligomers at the substoichiometric concentrations employed herein and as expected for a large antibody, they likely do not bind to cell membranes or displace protein misfolded oligomers. Moreover, the increases in both hydrophobicity and size induced by superstoichiometric concentrations of trodusquemine would be expected not to significantly change the cytotoxicity of the aggregates in the absence of the trodusquemine-induced displacement of oligomers; such an experiment could not be carried out given the intrinsic toxicity of trodusquemine at the concentrations necessary to observe these nonphysiologically relevant physiochemical changes [29]. Nonetheless, these findings show that the phenomena described herein extend beyond DesAb_18-24_ and DesAb_34-40_ to small molecules. 

Based on these results, we anticipate that it may become possible in future systematic studies to obtain more insights into the structure-toxicity relationship of oligomers with new DesAbs targeting disease-related proteins. Utilization of in vitro assays capable of predicting the toxicity of oligomers by probing their structural and physicochemical properties could facilitate enhanced throughput preclinical drug discovery studies by limiting in part the need of more expensive and time-consuming cell culture experiments.

## 4. Materials and Methods

### 4.1. Chemicals

Trodusquemine was synthesized as a hydrochloride salt at a purity greater than 97%, stored as a lyophilized powder [58,59] and solubilized in water to a final concentration of 10 mM. Stocks were stored at −20 °C until use.

### 4.2. Antibody Design, Expression and Purification

Antibodies were computationally designed as previously described [18,37]. Complementary peptides designed to bind specific epitopes were grafted onto the CDR loops of domain antibodies [18,37]. The expression and purification of the antibodies were carried out as previously described [18]. In brief, DesAb constructs were expressed and purified using pRSET-B vector in *E. coli* Rosetta-gami 2 (DE3, Merck Millipore, Burlington, MA, USA), cells were grown, harvested and lysed, and the antibodies (circa 18 kDa) were purified using a Ni^2+^-NTA Superflow column (Qiagen, Hilden, Germany) [18]. The His-tagged antibodies were eluted by increasing the concentration of imidazole, and were subsequently dialyzed thoroughly in PBS. Protein concentration was determined by absorbance at 280 nm [18]. DesAb aliquots were stored at −20 °C until use and never thawed more than once. The sequence of DesAb_18-24_ is MRGSHHHHHHGMASMTGGQQMGRDLYDDDDKDPKLEVQLVESGGGLVQPGGSLRLSCAASGFNIKDTYIGWVRRAPGKGEEWVASIYPTNGYTRYADSVKGRFTISADTSKNTAYLQMNSLRAEDTAVYYCAAGSVFVGTEAEEEAAAWGQGTLVTVSSGT and that of DesAb_34-40_ is MRGSHHHHHHGMASMTGGQQMGRDLYDDDDKDPKLEVQLVESGGGLVQPGGSLRLSCAASGFNIKDTYIGWVRRAPGKGEEWVASIYPTNGYTRYADSVKGRFTISADTSKNTAYLQMNSLRAEDTAVYYCAAGSIRETALLYEEEAAAWGQGTLVTVSSGT. 

### 4.3. Protein Expression and Chemical Kinetics

The recombinant Aβ(M1-40) peptide (sequence MDAEFRHDSGY EVHHQKLVFF AEDVGSNKGA IIGLMVGGVV), denoted as Aβ_40_, was expressed in the *Escherichia coli* BL21 Gold (DE3) strain (Stratagene, San Diego, CA, USA) and purified as described previously [19]. Samples for kinetic experiments were prepared using standard reagents and methods with a final buffer composition of 20 mM Tris, 100 mM NaCl, pH 7.4. The concentration of the purified protein was quantified as it eluted off the column by integrating the peak area of absorbance with ε_280_ = 1400 L mol^−1^ cm^−1^ [60]. 

For kinetic experiments, samples were prepared with monomeric Aβ_40_ at a concentration of 10 µM in the absence or presence of 0.5 µM, 1 µM, and 2 µM DesAbs. All samples for the kinetic measurements were supplemented with 0.2 mM ethylenediaminetetraacetic acid (EDTA) [29]. ThT was added from a 2 mM stock to give a final concentration of 20 µM. All samples were prepared in low-binding Eppendorf tubes, and samples were analyzed in a 96-well half area, low-binding, clear-bottom PEG coated plate (Corning 3881, Sigma-Aldrich, St. Louis, MO, USA). ThT fluorescence was monitored in triplicate per sample and measured using bottom-optics in a plate reader (Fluostar Omega or Fluostar Optima from BMG Labtech, Aylesbury, UK) with 440 and 480 nm excitation and emission filters, respectively. Aggregation was initiated by transferring the 96-well plate to the plate reader at 37 °C under quiescent conditions. The data shown are representative of three independent experiments that gave consistent results.

### 4.4. Preparation of the Stabilized Aβ_40_ Oligomers

First, 1 mg of lyophilized Aβ_40_ was solubilized overnight in 300 µL hexafluoroisopropanol (HFIP) at 4 °C. Solvent was evaporated with a gentle flow of nitrogen gas, and the protein was resuspended in DMSO to 2.2 mM. Two sonication steps of 10 min were preformed, after which the protein was diluted at a concentration of 100 µM in 20 mM sodium phosphate buffer, 200 μM ZnCl_2_, pH 6.9 for 20 h at 20 °C. Samples were centrifuged (15,000 rcf, 20 °C, 15 min) to a pellet and the supernatant was removed [42]. Oligomers were resuspended in buffer (20 mM Tris, 100 mM NaCl, pH 7.4) with thorough mixing. 

### 4.5. ANS Binding Measurements

First, 10 µM Aβ_40_ oligomers (monomer equivalents) were incubated (2 h, 20 °C) in the absence and presence of increasing concentrations of DesAbs (20 mM Tris, 100 mM NaCl, pH 7.4) at. Samples containing trodusquemine were incubated (1 h, 20 °C) at an oligomer concentration of 5 µM (20 mM Tris, 100 mM NaCl, pH 7.4) in the absence or presence of the small molecule at concentrations ranging from 5 µM to 50 µM. Subsequently, ANS was added to a final concentration of 30 µM or 15 µM, respectively (i.e., 3-fold excess dye in both cases). Emission spectra were recorded using a plate reader (BMG Labtech) with excitation at 380 nm. Duplicate samples are representative of three independent experiments that gave consistent results. All spectra were background subtracted to the buffer.

### 4.6. Turbidimetry Measurements

Samples from the ANS preparation were analyzed using a plate reader (BMG Labtech) with spectral scanning. Duplicate samples are representative of three independent experiments that gave consistent results. All spectra were background subtracted to the buffer.

### 4.7. SLS

First, 10 µM Aβ_40_ oligomers (monomer equivalents) were incubated for 2 h in the absence and presence of increasing concentrations of DesAbs (20 mM Tris, 100 mM NaCl, pH 7.4) at 20 °C. SLS measurements were performed using the Malvern Zetasizer Nano S instrument (Malvern Panalytical, Malvern, UK) with fixed parameters (attenuator 9, cell position 4.65 mm), equipped with a Peltier temperature controller (25 °C). A low volume (70 µL) disposable cuvette was used (BrandTech Scientific, Essex, CT, USA). Samples containing trodusquemine were incubated (1 h, 20 °C) at an oligomer concentration of 5 µM (20 mM Tris, 100 mM NaCl, pH 7.4) in the absence or presence of the small molecule at concentrations ranging from 5 µM to 50 µM.

### 4.8. Sedimentataion-Based Dot Blot Assays

Aβ_40_ oligomers were prepared at a concentration of 10 µM (monomer equivalents) and incubated in the absence or presence of 0.5, 1.0 and 2 µM DesAbs, as described above. For sedimentation measurements, 50 µL aliquots of samples were centrifuged (Eppendorf Centrifuge 5424 R, Hambrug, Germany) for 10 min at 13,000× *g* and 20 °C. Supernatant was removed and protein was thoroughly resuspended in 20 µL buffer prior to spotting the nitrocellulose membrane (2 µL per spot) (Whatman, pore size of 0.2 µm, GE Healthcare). Membranes were blocked with skim milk (Sigma-Aldrich) for 1 h, after which time the primary antibody 6E10 (803001, BioLegend, San Diego, CA, USA) was incubated at a 1:1000 dilution overnight at 4 °C. The Alexa488-conjugated secondary antibody (anti-mouse, Life technologies, Carlsbad, CA, USA) was subsequently incubated for 1 h at a 1:5000 dilution at 20 °C. Fluorescence was detected using a Typhoon Trio Imager (GE Healthcare, Chicago, IL, USA). Membranes were prepared in duplicate, and representative images are shown. ImageJ was used for quantifications.

### 4.9. Antibody Binding Dot Blot Assays

In order to assess the binding of the DesAbs, dot blots were prepared as follows: 4 μM solutions of DesAb were spotted (3.5 μL) onto a 0.2 μm pore size nitrocellulose membrane corresponding to total deposited antibody of 50, 25, 12.5 and 6.3 µg DesAb. The blots were blocked in PBS, 5% BSA overnight at 4 °C. Subsequently, they were incubated in solutions containing 5 μM Aβ_40_ oligomers in PBS overnight at 4 °C. Blots were then probed with the mouse monoclonal anti-amyloid β antibody (clone 6E10) (1:2000 dilution; Absolute Antibody Ltd., Oxford, UK) overnight at 4 °C, and then with goat anti-mouse IgG (H+L) secondary antibody and AF488 conjugate (1:5000 dilution; Life Technologies) for 2 h at 25 °C. 

### 4.10. Atomic Force Microscopy

Aβ_40_ oligomers (10 µM, monomer equivalents) were incubated in the absence and presence of 2 µM DesAb_34-40_, as previously described, and diluted by a factor of 20 before deposition atop an atomically flat mica surface. AFM samples preparation was carried out at room temperature by deposition of a 10 µL drop of solution deposited for 2 min to a freshly-cleaved mica surface. Salt was washed with water, and samples were dried by the gentle flow of nitrogen and stored in a sealed container until imaging using a Park NX10 AFM (Park Systems, Suwon, Korea) with scan rates < 0.4 Hz and PPP-NCHR cantilevers with an 8 nm nominal radius (Nanosensors, Neuchatel, Switzerland). To visualize the trodusquemine-oligomer complex, Aβ_40_ oligomers (5 µM, monomer equivalents) were incubated in the absence and presence of 25 µM trodusquemine for 1 h as previously described. These samples were diluted in buffer by a factor of five and subsequently sprayed at 100 µL/h for 1 min. at room temperature using a recently described microfluidic device [49] atop an atomically flat mica surface. Samples were stored in a sealed container until imaging using a JPK Nanowizard2 AFM with scan rates < 0.3 Hz and a silicon tip with a 10 nm nominal radius (2 N m^−1^, MikroMasch, Wetzlar, Germany). For all samples, standardized scanning conditions were established and maintained while measuring the samples, such that a phase change on the order of ≈Δ20° upon the interaction of the tip with the sample was not exceeded; changes corresponding to dissipated energy during the sample–tip interactions in excess of this threshold can lead to artifacts in the height quantification caused by deformations induced in the sample by the tip [29,45,61]. Three-dimensional maps were flattened using the SPIP (Image Metrology, Horsholm, Denmark) software, and the cross-sectional height of unique aggregates was compared [47,61].

### 4.11. Neuroblastoma Cell Cultures

Human SH-SY5Y neuroblastoma cells (Sigma-Aldrich, origin from A.T.C.C., Manassas, VA, USA) were cultured in DMEM, F-12 HAM with 25 mM HEPES and NaHCO_3_ (1:1) and supplemented with 10% FBS, 1 mM glutamine and 1.0% antibiotics. Cell cultures were maintained in a 5% CO_2_ humidified atmosphere at 37 °C, and grown until they reached 80% confluence for a maximum of 20 passages [62,63]. Cell lines were authenticated, and tested negative for mycoplasma contaminations. To count the cells, old medium was removed from the flask and the cells were detached using trypsin (Thermo Fisher, Waltham, MA, USA). An equal volume of medium was used to neutralize the trypsin once the cells were fully in suspension, as monitored under an inverted microscope. Cells were centrifuged gently to a pellet (1500 rpm, 5 min, 25 °C) and resuspended in fresh medium. Then, 10 µL cells were mixed with 10 µL trypan blue stain to stain dead cells (0.4%, Thermo Fisher) and counted using the Countess II (Thermo Fisher).

### 4.12. MTT Reduction Assay

Aβ_40_ oligomers (5 or 10 µM in monomer equivalents, as indicated) were incubated in the absence and presence of 20:1 to 5:1 molar ratios of the designed antibodies in cell culture medium for 1 h at 37 °C under shaking conditions, and then added to the cell culture medium of SH-SY5Y cells seeded in 96-well plates for 24 h. The 3-(4,5-dimethylthiazol-2-yl)-2,5-diphenyltetrazolium bromide (MTT) assay was performed as previously described [63,64].

### 4.13. Statistics and Data Availability

Statistical analyses were performed in GraphPad Prism 8 (GraphPad Software, La Jolla, CA, USA). All error bars denote standard error of the mean (SEM). One-way ANOVA was used for multiple comparisons as a function of increasing concentration of DesAbs relative to oligomers incubated in buffer or cell culture medium (with Bonferroni’s multiple comparisons test). Comparisons between untreated cells and oligomer-exposed cells were performed using an unpaired, two-tailed Student’s *t*-test. *p* < 0.05 was accepted as statistically significant. Data are available from the authors upon request.

## Supplementary Materials

Supplementary materials can be found at https://www.mdpi.com/1422-0067/21/12/4542/s1.

## Abbreviations

Aβ	amyloid-β peptide
Aβ_40_	40-residue form of the amyloid-β peptide
Aβ_42_	42-residue form of the amyloid-β peptide
AD	Alzheimer’s disease
AFM	atomic force microscopy
ANS	8-anilinonapthalene-1-sulfonic acid
DesAb	designed antibody
HFIP	hexafluoroisopropanol
MTT	3-(4,5-dimethylthiazol-2-yl)-2,5-diphenyltetrazolium bromide
SEM	standard error of the mean
SLS	static light scattering
ThT	thioflavin T
TRO	trodusquemine
