# Supplementary material for: Rationally Designed Antibodies as Research Tools to Study the Structure–Toxicity Relationship of Amyloid-β Oligomers
Source: Int J Mol Sci. 2020 Jun 25;21(12):4542. doi: 10.3390/ijms21124542 (PMC7352524; doi:10.3390/ijms21124542)
Supplement: Supplementary file 1 [file ijms-21-04542-s001.pdf]

Supporting information for:

Rationally Designed Antibodies as Research Tools to Study the Structure–Toxicity Relationship of Amyloid-β Oligomers

Ryan Limbocker [1,2,*], Benedetta Mannini [1], Rodrigo Cataldi [1], Shianne Chhangur [1], Aidan K. Wright [2], Ryan P. Kreiser [2], J. Alex Albright [2], Sean Chia [1], Johnny Habchi [1], Pietro Sormanni [1], Janet R. Kumita [1], Francesco S. Ruggeri [1], Christopher M. Dobson [1], Fabrizio Chiti [3], Francesco A. Aprile [1,†,*] and Michele Vendruscolo [1,*]

[1] Centre for Misfolding Diseases, Department of Chemistry, University of Cambridge, Cambridge CB2 1EW, UK; bm475@cam.ac.uk (B.M.); rlc67@cam.ac.uk (R.C.); sc2066@cam.ac.uk (S.C.); skrc2@cam.ac.uk (S.C.); jh884@cam.ac.uk (J.H.); ps589@cam.ac.uk (P.S.); jrk38@cam.ac.uk (J.R.K.); fsr26@cam.ac.uk (F.S.R.); cmd44@cam.ac.uk (C.M.D.)

[2] Department of Chemistry and Life Science, United States Military Academy, West Point, NY 10996, USA; aidan.wright@westpoint.edu (A.K.W.); ryan.kreiser@westpoint.edu (R.P.K.); james.albright@westpoint.edu (J.A.A.)

[3] Department of Experimental and Clinical Biomedical Science, University of Florence, 50134 Florence, Italy; fabrizio.chiti@unifi.it

* Correspondence: ryan.limbocker@westpoint.edu (R.L.); f.aprile@imperial.ac.uk (F.A.A.); mv245@cam.ac.uk (M.V.)

† Present address: Department of Chemistry, Molecular Science Research Hub, Imperial College London, London W12 0BZ, UK

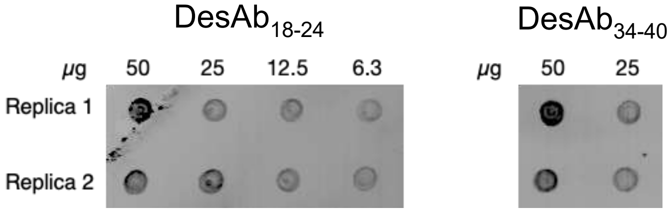

Supplementary Figure S1. Binding of the DesAbs to Aβ_{40} oligomers. Solutions of DesAb to various degrees of dilutions were spotted (3.5 µL) on a 0.2 µm pore size nitrocellulose membrane to a total amount of 50, 25, 12.5 and 6.3 µg DesAb and subsequently incubated overnight in solutions containing 4 µM (monomer equivalents) Zn^{2+}-stabilized Aβ_{40} oligomers. After washing away unbound protein, the monoclonal anti-amyloid β antibody 6E10 was used to recognize Aβ_{40} bound to DesAbs.

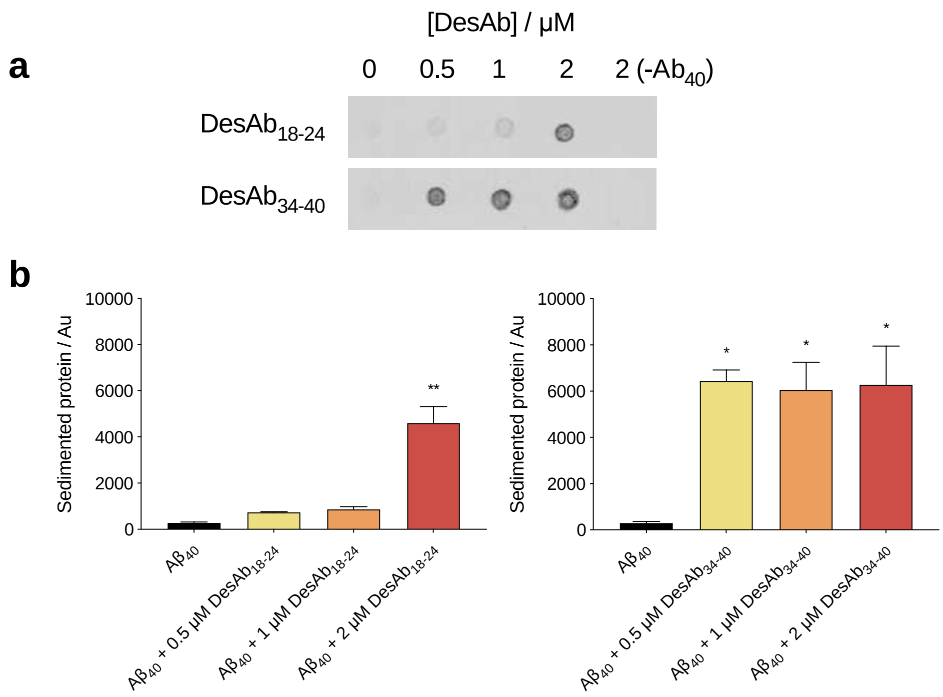

Supplementary Figure S2. The DesAbs increase the sedimentation propensity of the stabilized $A\beta_{40}$ oligomers. The proportion of oligomers able to sediment was measured using a dot-blot assay for $A\beta_{40}$ oligomers incubated at a concentration of 10 μM in the absence (black) or presence of 0.5 μM (yellow), 1 μM (orange) and 2 μM (red) of $DesAb_{18-24}$ (left) and $DesAb_{34-40}$ (right). Note that 2 μM of both DesAbs in the absence of oligomers were not visible in the assay. Data were quantified for pixel intensity in ImageJ (gel analysis). Error bars denote SEM of duplicate technical replicates. Data shown are representative of two experiments that gave consistent results. The symbols * and ** indicate $p < 0.05$ and 0.01, respectively.

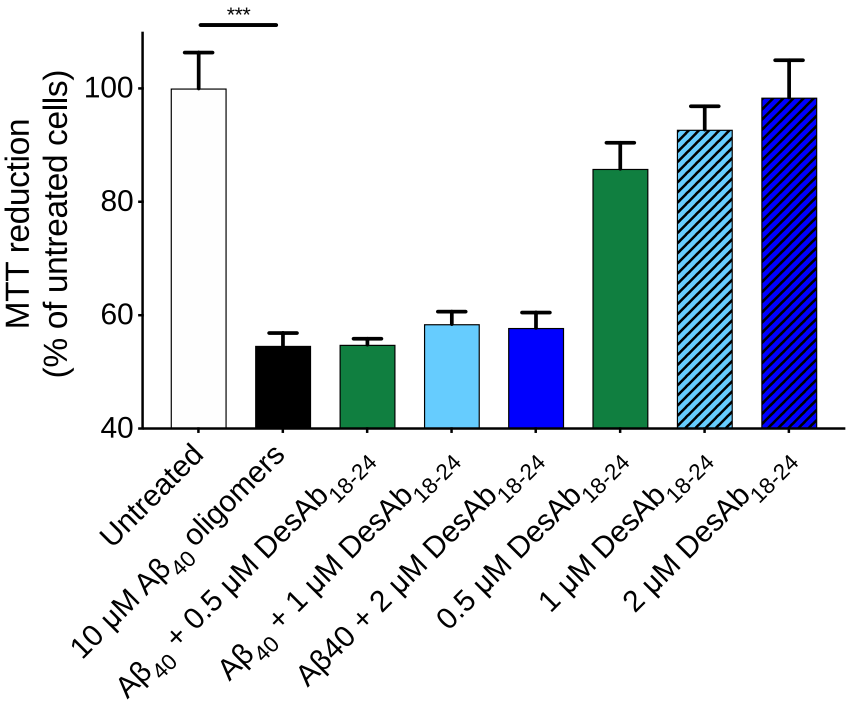

Supplementary Figure S3. The DesAbs do not alter oligomer toxicity when tested at higher concentrations of oligomers and antibodies. Stabilized $A\beta_{40}$ oligomers were resuspended in the cell culture medium at a concentration of 10 μM and incubated without (black plain bar) or with increasing concentrations (0.5, 1 and 2 μM) of $DesAb_{18-24}$ for 1 h at 37 °C and then added to the cell culture medium of SH-SY5Y cells for 24 h (colored plain bars). The cells were also treated with corresponding concentrations of the antibodies pre-incubated in the absence of oligomers (dashed bars). Error bars indicate SEM of seven replicates. The symbol *** indicates $p < 0.001$ (unpaired, two-tailed Student's t-test). All conditions containing both antibodies and oligomers were not significantly different in comparison to cells treated with oligomers alone (one-way ANOVA using Bonferroni's multiple comparisons relative to cells treated with oligomers alone).

Supplementary Table S1. Predicted and experimentally observed cellular viabilities for oligomers in the presence of the DesAbs. The listed and experimentally measured wavelengths of maximum ANS fluorescence and Rayleigh ratios from the SLS measurements were used to predict the change in MTT reduction caused by $A\beta_{40}$ oligomers in the absence and presence of increasing concentrations of DesAbs using a previously described formula [30]. The corresponding experimentally observed changes in MTT reduction are also shown.

Oligomer:Antibody Ratio	Wavelength of Max ANS Fluorescence (nm)	Rayleigh Ratio	Predicted Change in Viability	Observed Change in Viability
$A\beta_{40}$	478.5	$2.32 \cdot 10^{-4}$	0%	0.0 ± 4.7%
$A\beta_{40}$ + 20:1 $DesAb_{18\text{-}24}$	478	$2.95 \cdot 10^{-4}$	0.4%	–1.2 ± 3.9%
$A\beta_{40}$ + 10:1 $DesAb_{18\text{-}24}$	478	$3.30 \cdot 10^{-4}$	1.8%	4.5 ± 3.6%
$A\beta_{40}$ + 5:1 $DesAb_{18\text{-}24}$	477	$3.91 \cdot 10^{-4}$	0%	–2.4 ± 4.1%
$A\beta_{40}$ + 20:1 $DesAb_{34\text{-}40}$	477	$3.16 \cdot 10^{-4}$	–2.9%	0.5 ± 3.6%
$A\beta_{40}$ + 10:1 $DesAb_{34\text{-}40}$	477	$3.57 \cdot 10^{-4}$	–1.3%	2.9 ± 4.2%
$A\beta_{40}$ + 5:1 $DesAb_{34\text{-}40}$	476	$4.32 \cdot 10^{-4}$	–2.5%	–0.9 ± 3.6%
